# Toward objective monitoring of Parkinson's disease motor symptoms using a wearable device: wearability and performance evaluation of PDMonitor^®^

**DOI:** 10.3389/fneur.2023.1080752

**Published:** 2023-05-16

**Authors:** Angelo Antonini, Heinz Reichmann, Giovanni Gentile, Michela Garon, Chiara Tedesco, Anika Frank, Bjoern Falkenburger, Spyridon Konitsiotis, Konstantinos Tsamis, Georgios Rigas, Nicholas Kostikis, Adamantios Ntanis, Constantinos Pattichis

**Affiliations:** ^1^Parkinson and Movement Disorders Unit, Study Center for Neurodegeneration (CESNE), Department of Neuroscience, University of Padua, Padua, Italy; ^2^Department of Neurology, University Hospital Carl Gustav Carus and Carl Gustav Carus Faculty of Medicine, Technische Universitat Dresden, Dresden, Germany; ^3^German Center for Neurodegenerative Diseases (DZNE), Dresden, Germany; ^4^Department of Neurology, University Hospital of Ioannina and Faculty of Medicine, School of Health Sciences, University of Ioannina, Ioannina, Greece; ^5^PD Neurotechnology Ltd., Ioannina, Greece; ^6^Department of Computer Science and Biomedical Engineering Research Centre, University of Cyprus, Nicosia, Cyprus

**Keywords:** Parkinson's disease, telemonitoring, wearable devices, digital health, automatic ambulatory monitoring, inertial measurement unit sensors

## Abstract

Parkinson's disease (PD) is characterized by a variety of motor and non-motor symptoms. As disease progresses, fluctuations in the response to levodopa treatment may develop, along with emergence of freezing of gait (FoG) and levodopa induced dyskinesia (LiD). The optimal management of the motor symptoms and their complications, depends, principally, on the consistent detection of their course, leading to improved treatment decisions. During the last few years, wearable devices have started to be used in the clinical practice for monitoring patients' PD-related motor symptoms, during their daily activities. This work describes the results of 2 multi-site clinical studies (PDNST001 and PDNST002) designed to validate the performance and the wearability of a new wearable monitoring device, the PDMonitor^®^, in the detection of PD-related motor symptoms. For the studies, 65 patients with Parkinson's disease and 28 healthy individuals (controls) were recruited. Specifically, during the Phase I of the first study, participants used the monitoring device for 2–6 h in a clinic while neurologists assessed the exhibited parkinsonian symptoms every half hour using the Unified Parkinson's Disease Rating Scale (UPDRS) Part III, as well as the Abnormal Involuntary Movement Scale (AIMS) for dyskinesia severity assessment. The goal of Phase I was data gathering. On the other hand, during the Phase II of the first study, as well as during the second study (PDNST002), day-to-day variability was evaluated, with patients in the former and with control subjects in the latter. In both cases, the device was used for a number of days, with the subjects being unsupervised and free to perform any kind of daily activities. The monitoring device produced estimations of the severity of the majority of PD-related motor symptoms and their fluctuations. Statistical analysis demonstrated that the accuracy in the detection of symptoms and the correlation between their severity and the expert evaluations were high. As a result, the studies confirmed the effectiveness of the system as a continuous telemonitoring solution, easy to be used to facilitate decision-making for the treatment of patients with Parkinson's disease.

## 1. Introduction

Parkinson's disease (PD) is a neurodegenerative disorder with a high prevalence among those aged ≥45 years (572 patients per 100, 000 people) (1). It is characterized by motor and non-motor symptoms, with a progressively worsening course. The main motor manifestations of the disease are bradykinesia, rigidity and resting tremor, with accompanying gait impairment and reduced manual dexterity (2). Non-motor symptoms include autonomic nervous system disorders, dementia, as well as neuropsychiatric disorders (3–5). To date, treatment is based on dopamine replacement drugs but there are numerous biological strategies under development including active and passive immunization aimed at testing disease modification (2, 6). In the early stages, drug treatment results in sustained benefits and improves quality of life throughout the day. However, as disease progresses, levodopa effects shorten, and patients experience motor and non-motor fluctuations, as well as, in some occasions, levodopa induced dyskinesia (LiD) and freezing of gait (FoG). To optimize and personalize the treatment strategy, it is necessary to accurately monitor their symptoms, as they vary widely from day-to-day, and also differ significantly between different patients (7). Rating scales for clinical evaluation, internet-based tools, completed by physicians, and diaries/questionnaires, completed by patients and caregivers have been developed to improve disease assessment of the clinical features of the disease (8, 9). However, the information from the diaries is often unclear and the limited time of the neurological assessment, during patient encounters, does not provide sufficient information to accurately determine the severity of symptoms that patients experience in their daily living and their own environment. This often results in underestimating or overestimating the symptoms of the disease and could lead to sub-optimal therapeutic interventions (10).

To address this issue, sensor-based systems have been developed for the quantitative evaluation of motor symptoms' severity, and some of them have been specifically designed for tracking PD symptoms (11–14). The idea of telemedicine is not new (15), but during the last 20 years, technological advancements and enhancement of telecommunication infrastructure, have made the accurate remote monitoring of patients with diverse disorders, such as PD, possible (16, 17). For neurodegenerative diseases, affecting both motor and cognitive functions, technological health services have emerged as useful tools for tackling the challenge of patient-physician contact, in cases where patients' visits to medical centers are laborious (18). Especially during the COVID-19 pandemic, a number of restrictions were imposed, forcing patients, caregivers and healthcare professionals toward limiting their interactions, thus encouraging the use of healthcare practices supported by electronic processes (eHealth) (19). This practice resulted in better healthcare technologies and related services, and led to their widespread adoption (20–22). Apart from remote delivery of health services to overcome barriers in communication and transportation, telemedicine in PD also involves accurate objective symptom detection, monitoring and improvement of follow-up care (18, 23). Different telemedicine modalities have been successfully employed in patients' care, including:

1. virtual visits via video conferencing (24),
2. non-motor symptom assessment/treatment via phone (25),
3. monitoring through wearable devices (23),
4. health applications on mobile phones (mHealth) (26),
5. virtual reality rehabilitation (27) and
6. online speech assessment and rehabilitation (28).

Telemedicine technology enables a patient-centric approach and has been proven to be reliable in the management of specific disease aspects, having comparable results with current medical practice (29, 30). Furthermore, the cost-effectiveness of telemedicine in PD has been analyzed in several studies that show considerable resource savings stemming from technology enhanced and home-based monitoring (31–34). Of course, disadvantages do exist, since telemedicine may limit the diagnostic ability and the patient-physician relationship, however, healthcare technology devices are currently recommended for use in response to existing clinical needs and have been integrated in the PD multidisciplinary care (19, 35). Wearable devices are the spearhead of eHealth modalities in PD. The reason for this lies mainly in the fact that symptoms' fluctuations in patients with PD cannot be reliably addressed with the current clinical limited assessment, while wearables can offer prolonged objective measurements of motor symptoms (11, 23).

Most of these wearable systems are based on inertial sensors that consist of accelerometers and gyroscopes. Griffiths et al. (36) presented a wearable system composed of a single sensor in the form of a wrist-worn watch and reported high accuracy in the detection of bradykinesia and dyskinesia, compared to clinical examination (37). The system was further validated in subsequent studies for fluctuation detection (38, 39), impairment in activities of daily living (40) and overall therapeutic management of patients with PD (41). However, since this system is worn on a single wrist, it can only measure a subset of PD symptoms, and specifically those related to that limb. Thus, gait impairment, dyskinesia, as well as freezing of gait, cannot be detected as they would require additional sensors (42–44). As a result, the presented system lacked the ability to extract information comparable with patient diaries, or more importantly, with a full neurological examination. Ferreira et al. (37) introduced another system based on wearable sensors and accompanied by a mobile app, for which they evaluated its wearability and usability (45). The clinical validity of the system was also evaluated and high accuracy was reported in leg dyskinesia assessment and fluctuation detection, without any report about the detection of other parkinsonian symptoms (13). For the detection of specific symptoms, other sensors have been developed as well (12, 46). A recent systematic review described wearable solutions developed for PD and summarized their advantages and disadvantages (47). Although the advancements in telemonitoring solutions are significant, monitoring technologies for PD haven't yet gained wide acceptance among physicians, patients and caregivers. The reason lies in the lack of adequate evidence for validating their clinical utility in specific conditions, including their use in the selection of suitable patients for invasive therapies (48–50). During the last couple of years, a paradigm shift in the monitoring of patients with PD is taking place. But, in order to be successful, it needs further support that can only be provided by the development of devices that can accurately monitor parkinsonian symptoms and evaluate their fluctuations in the long term. Perhaps the most important aspect of this process is to prove that the output of the monitoring devices is accurate, thus extensive validation is necessary (23). Preliminary data on acceptability originating from patients of these systems are encouraging and have helped define outcome measures for clinical studies (51).

To that end, the PDMonitor^®^ system (PD Neurotechnology Ltd.) was developed for the continuous monitoring of Parkinson's disease symptoms, designed to be used by patients in their own environment. The PDMonitor^®^ is an innovative device consisting of five wearable sensors, to be worn on the trunk and then limbs, and is able to detect remotely most motor manifestations of PD, including the daily activity of patients in their home. It is also intended for long term follow-up monitoring of each patient with the goal of objectively assessing the course of the disease. The aim of this work was to use complementary data from 2 multi-site clinical studies, described in Section 2.3, as a first systematic validation of the usability and the performance detected of the PDMonitor^®^ system in the identification, quantification and monitoring of PD motor symptoms. More specifically, the main questions this works aimed to answer, were:

- *Is the device feasible to be used by patients and caregivers without supervision?*
- *Is the device reliable when compared to expert assessment of PD symptoms?*

## 2. Materials and methods

Section 2.1 describes the body-worn system used for the evaluation of PD motor symptoms. Section 2.2 briefly describes the methods and algorithms used by the system. Section 2.3 briefly describes the data collection used for the initial algorithm verification, as well as the studies performed for the validation of the device.

### 2.1. The PDMonitor^®^ system

**Figure 1:**
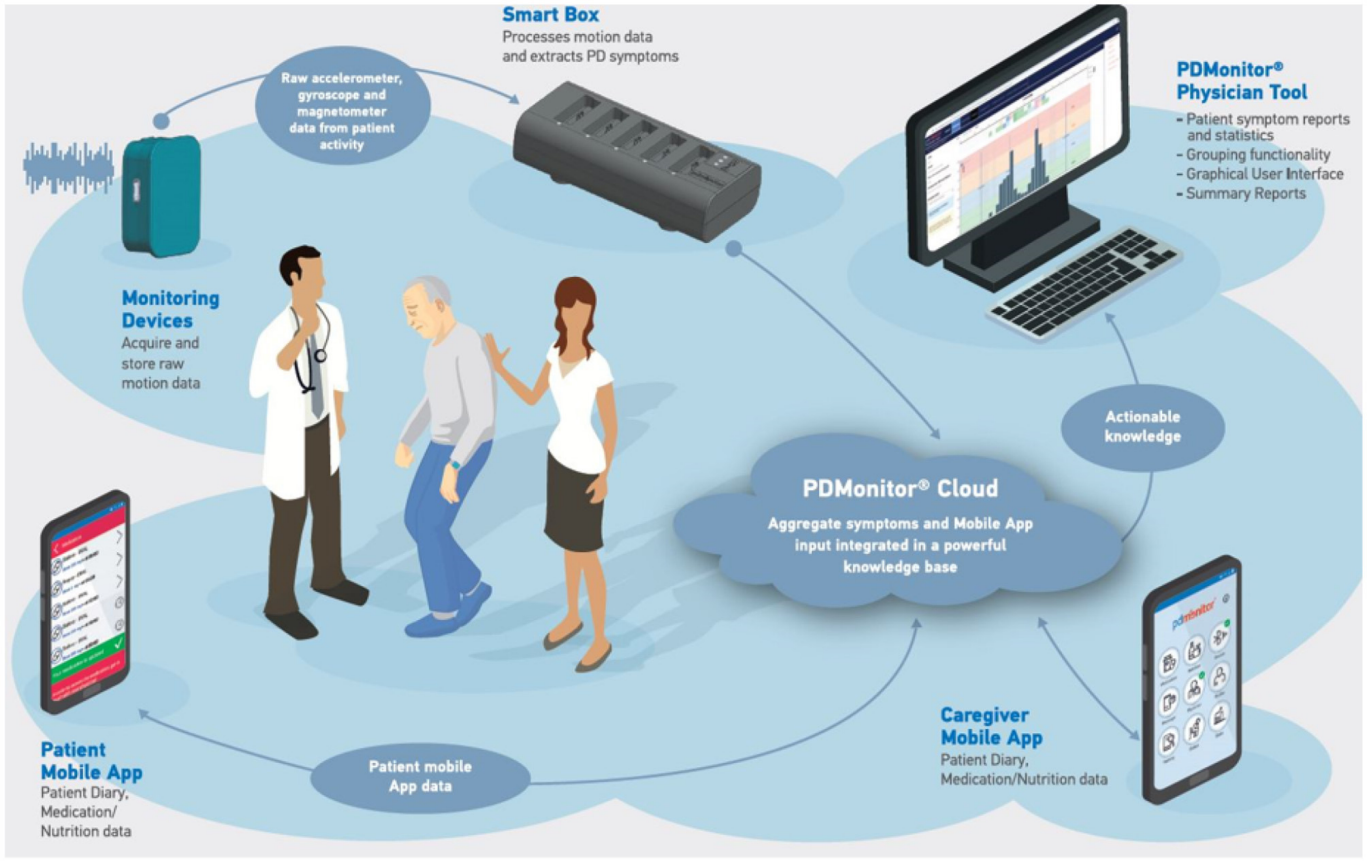
The PDMonitor^®^ ecosystem.

The PDMonitor^®^ system developed by PD Neurotechnology^®^ Ltd. is a class IIa CE-marked medical device, intended to be used by patients diagnosed with PD, for continuous home monitoring. The system is comprised of a base, a set of monitoring devices, a set of mounting accessories, a mobile application, a physician web dashboard and a cloud service. The PDMonitor^®^ provides an ecosystem (Figure 1) enabling long term continuous remote monitoring of patients with Parkinson's disease (PwPs).

**Figure 2:**
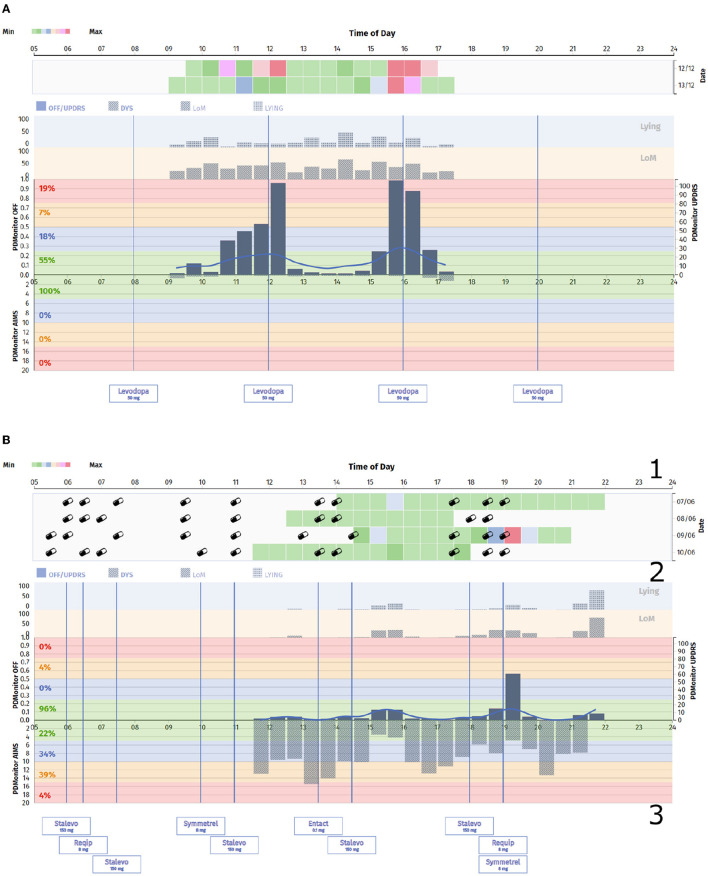
**(A)** PDMonitor^®^ OFF/Dyskinesia chart for a patient with clear fluctuations. **(B)** PDMonitor^®^ OFF/Dyskinesia chart for a patient with significant dyskinesia. In this report the different areas of interest have been marked. Specifically, the area 1 illustrates the severity of a symptom for a 30-min interval, including medication and nutrition information, the area 2 presents a chart with the average symptom intensity for any time of the day, while the area 3 lists the medication the patient receives.

**Figure 3:**
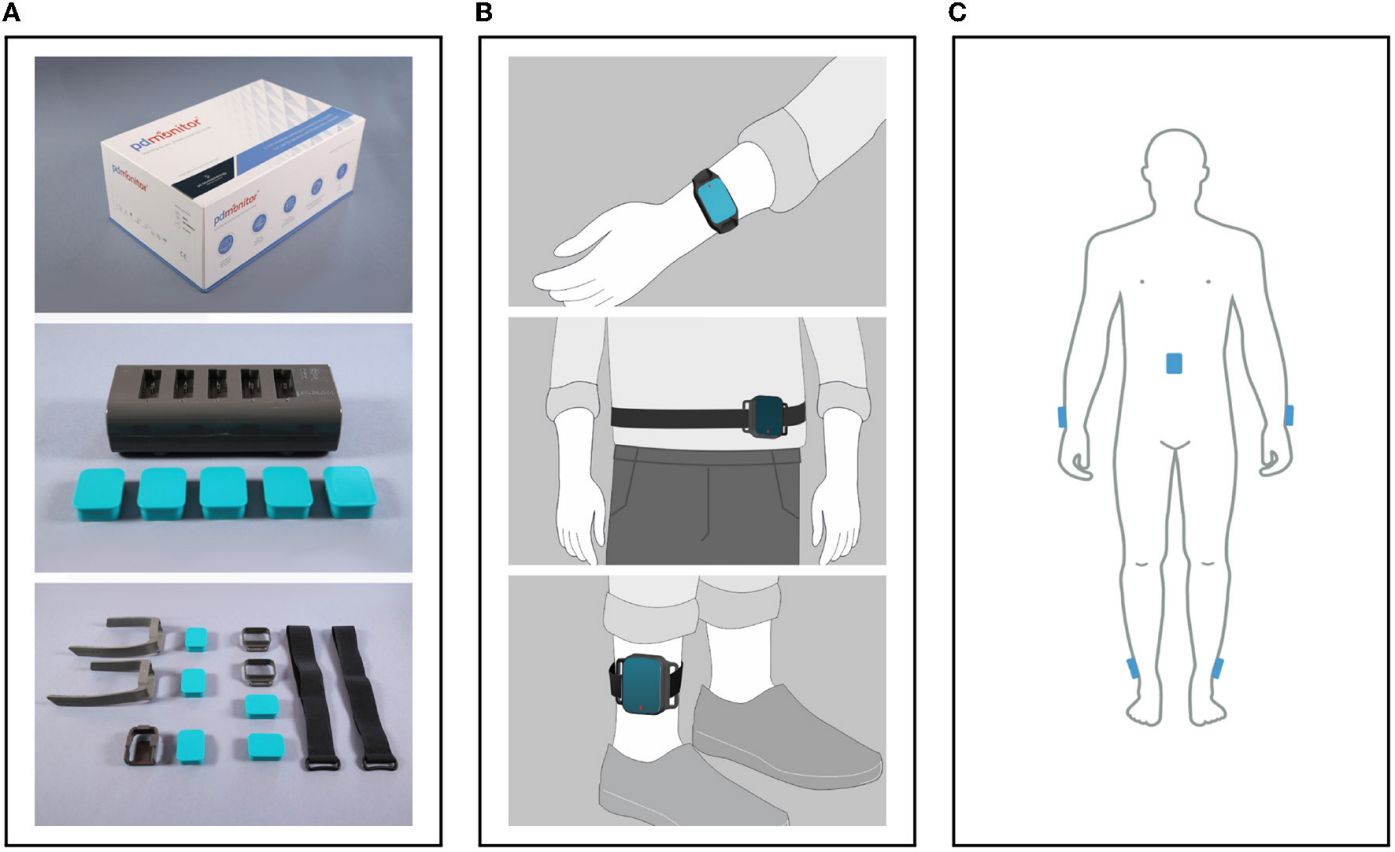
**(A)** The PDMonitor^®^ box, docking station, monitoring devices and accessories. **(B)** The PDMonitor^®^ monitoring devices' placement on the wrists, torso and ankles. In the middle image, the waist sensor has been placed to the waist with a velcro band and a StrapFrame, but there is also the option to be mounted on a belt using a ClipFrame accessory. **(C)** The placement of all monitoring devices on the appropriate body position at the same time.

Physicians have full access to patients' symptom reports at any time, with comprehensive information about almost all PD-related motor symptoms *via* the physicians' web dashboard. Two different patient cases from the study, with different symptoms, as they appear in the web portal, are presented in Figure 2. The PDMonitor^®^ report consists of a heatmap, illustrating the severity of a symptom for a 30-minute interval and a chart with the average symptom intensity for any time of day. The reports also provide the medication schedule and the actual medication intake (as well as nutrition information) reported by the patient via the PDMonitor^®^ mobile application. Although the web dashboard is the default way of accessing the outputs of the system, if there is a need for direct access to the raw IMU data, then one would need to contact PD Neurotechnology Ltd. beforehand, i.e., before the patient uses the device. The components of the PDMonitor^®^ system are the following (Figure 3A):

1. The PDMonitor^®^ SmartBox, used to collect, process and upload data to the cloud. The SmartBox also acts as a docking station for charging the wearable sensing devices (Monitoring Devices) after they have been used. The SmartBox has a size of 170 × 80 × 17mm and a weight of ≈280g.
2. Five wearable sensing monitoring devices, used to collect movement data. Each monitoring device has a size of 41 × 30.6 × 12.85mm, a weight of ≈16g and contains a 9-degree inertial measurement unit (IMU) sensor (accelerometer, gyroscope and magnetometer), the LSM9DS1 from ST Microelectronics. The monitoring devices record data with a sampling frequency of 59.5Hz, which they store internally, until they are docked to the SmartBox, at which point the data are transferred and uploaded to the Cloud. The LSM9DS1 has a linear acceleration full scale of ±2/±4/±8/±1g, a magnetic field full scale of ±4/±8/±12/±16gauss and an angular rate of ±245/±500/±2000dps.
3. PDMonitor^®^ accessories (i.e., ClipFrame, StrapFrame, Wristband and Velcro straps), used to attach the monitoring devices to the patient's body, and more specifically, near the ankles, wrists and waist. Regarding the ankles, the monitoring devices are attached to the lateral compartment of the leg, slightly above the ankle, whereas the wrist monitoring devices are attached to the posterior compartment of the forearm around the wrist, much like a watch. The waist monitoring device is placed near the anterior midline of the body at the height of the waist. The waist sensor can be mounted, either with a velcro band paired with a StrapFrame, or with a ClipFrame, based on the patient's preferences. The proper device placement is presented in Figures 3B, C.

Each PDMonitor^®^ monitoring device produces raw measurements from its embedded IMU sensor. Subsequently, all 5 are synchronized and their data are uploaded to the Cloud when docked to the SmartBox. Then, the symptom evaluation process transforms the raw IMU signals from all monitoring devices to a unique set of movement features, which are in turn converted to symptom estimations for 30-minute windows, correlated to UPDRS or other relevant scales' items. The final movement items estimations are the output of the PDMonitor^®^ device to the cloud. The PDMonitor^®^ symptom evaluation involves data analysis with digital signal processing techniques, feature extraction algorithms, and machine learning. The final outcome is the automated quantification of basic daily activities (walking, resting/sitting, lying), main parkinsonian motor symptoms (tremor, bradykinesia, gait and balance impairments), and the most important motor complications associated with the antiparkinsonian therapy (ON/OFF fluctuations, LiD and FoG). Based on the system's intended use, the 5 monitoring devices must be worn by the patients during their waking hours, and then docked for data transfer and recharging during the rest of the day. However, the sensors have a battery duration of up to 50 h and thus this is considered the maximum recording duration.

Although there are 5 monitoring devices to be attached to a patient's body, PDMonitor^®^ is easy to use, due to its ability to automatically identify the placement of each sensing device on the waist and limbs (52). As a result, the users (patients and/or caregivers), do not need to match each sensor, individually, to a corresponding body position, thus, reducing both the time necessary for mounting the sensors and the probability of user error. Moreover, PDMonitor's^®^ sensor-mounting accessories (i.e., the Wristbands, StrapFrames and ClipFrames) act as active measures against inappropriate use (i.e., placing them in a wrong orientation). However, caution by the users remains a prerequisite to place, both the wrist, and the ankle sensors facing outwards, to prevent misidentification between the left and right limbs. An inwards placement of the limb monitoring devices would be improbable, given the awkward and uncomfortable nature of this configuration, especially for the wrist sensors.

### 2.2. The PDMonitor^®^ algorithms

The PDMonitor^®^ algorithms were initially designed, and preliminary developed, during the PERFORM project (53–55). Subsequently, they were further/mainly developed and verified in a Pilot study performed at the University Hospital of Ioannina (see Section 2.3.3).

**Figure 4:**
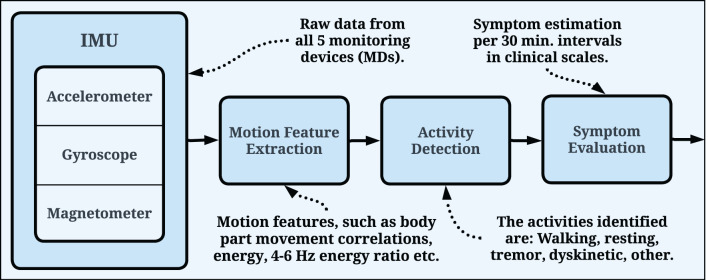
General pipeline used by the PDMonitor^®^ algorithms. The raw IMU signals are used for motion feature extraction, which are, in turn, utilized for symptom evaluation. The evaluation is generated every 30 min and the symptoms are presented in relevant clinical scales.

The symptom evaluation process is similar for all PDMonitor^®^ symptom assessment algorithms (Figure 4). More specifically, all devices collect IMU sensor raw measurements (accelerometer, gyroscope, magnetometer). Each sensor has three axes (X, Y, Z), therefore it is a 9-degree measurement system. The PDMonitor^®^ symptom evaluation process transforms the raw IMU signals from all monitoring devices to a unique set of movement features, which are in turn converted to UPDRS, or other clinical scales' items, estimated in 30-minute windows. The first step in the overall PDMonitor^®^ symptom evaluation methodology is activity detection as described in Section 2.2.1. After activity detection, symptom-specific processing is used to address the challenging task of detecting, quantifying and assessing each of the cardinal PD motor symptoms. Machine learning is mainly applied in this step in order to discriminate different types of body movement (walking, normal activity, leg tremor and dyskinesia) and in every case a different kind of symptom assessment takes place. In the following sections, the methods and algorithms used in the PDMonitor^®^ system will be briefly described, mainly focusing on activity and posture detection, dyskinesia, bradykinesia, gait, tremor and ON/OFF fluctuations. Due to space limitations, algorithms are not presented in detail. Nonetheless, the main features used by each method are described.

#### 2.2.1. Activity detection

The PDMonitor^®^ symptom evaluation process for symptom detection and severity assessment follows a hierarchical approach. The main idea is to first identify “regions of interest” based on the activity, where each symptom can be evaluated with high accuracy. For example, regions in the signal identified as rest are used to detect resting tremor, while regions that include climbing of stairs are used to detect gait impairment. This requires accurate activity detection algorithms. The main activities identified were: Walking, Resting, Tremor, Dyskinetic and Other. Walking pertains to free walking (excluding stair climbing), however the patients that took part in the generation of the dataset, were free to move without restrictions. Hence, the dataset itself, and as a result the symptom evaluation process, take into account numerous other activities (i.e., rehabilitation activities), which were not explicitly annotated. Those activities are included in the “Other” category.

The PDMonitor^®^ activity detection is based on motion features extracted from all body parts, as well as from both time (signal energies, average values, standard deviations, jerk, correlation of signals from different body parts etc.) and frequency domain (energies of gyroscope signals within different frequency bins). The objective of the activity detection is to evaluate different activities based on the quantification of body movement, movement coordination (walking is a coordinated body motion whereas dyskinesia is not) and posture (by discriminating between standing, sitting and lying). In total, over 140 features are extracted. A wrapper feature selection method (56) is applied to identify the best feature set for Bayes classification. The activity detection method was developed and verified with data from the pilot study. A Naive Bayes classifier is applied using a leave-one-out technique, which minimizes the risk of overfitting and bias. With this approach, PDMonitor^®^ managed to identify the different body movements with high accuracy (>90%). The identification of each activity spawns further analysis for different symptoms and motor characteristics. Gait disturbances are evaluated exclusively during the “Walking” activity, dyskinesia severity is assessed during the “Dyskinetic” activity, whereas (wrist) tremor and arm bradykinesia are assessed during the “Resting” or “Other” activities. The general pipeline used by the PDMonitor^®^ algorithms is presented in Figure 4.

#### 2.2.2. Dyskinesia

The dyskinesia evaluation algorithm requires activity detection to be implemented first. Dyskinesia severity is better assessed while resting, therefore walking regions are excluded. The first step is to find dyskinetic regions in 5-minute window intervals. In a 5-minute window, the initial detected activity is combined with motion features from all body parts into a new feature vector enabling the detection of dyskinesia and the assessment of its severity.

#### 2.2.3. Bradykinesia

The PDMonitor^®^ method for the detection and assessment of bradykinesia is based on the evaluation of a patient's movement speed. However, to assess movement capacity, actual movement must occur and be detected. Therefore, bradykinesia evaluation starts with the detection of specific movements and the estimation of their speed. Movements that are slower than those calculated for the control group are considered as bradykinetic movements. The percentage of the bradykinetic movements for a 30-minute window is the so-called “PDMonitor^®^ bradykinesia score” which is significantly correlated with the UPDRS score of arm bradykinesia (items 23, 24 and 25).

#### 2.2.4. Gait

The gait assessment requires the identification of walking regions and the detection of individual steps. The main parts of the method are: signal acquisition and filtering, activity detection, consecutive candidate walking regions' merging, steps detection, gait features extraction and gait impairment score extraction based on gait features. The detection of gait is based on the activity detection method. The basic window used for activity detection is 4 s. Consecutive windows classified as “Walking” are merged into larger walking regions in order to improve the statistical estimation of gait parameters. After walking region detection and merging, a step detection procedure is applied. Three peaks are identified for each step: Terminal Contact (TC), corresponding to heel off, Max Rotational Speed (RS), corresponding to mid-stance and Initial Contact (IC), corresponding to heel strike. Then, a number of features are estimated based on the detected peaks for each step. A number of gait features are extracted (shanks' sagittal range of movement, cadence, swing time, swing time variability among others) and combined in order to build a linear model with the purpose of translating gait features to the gait corresponding item of the UPDRS scale. The feature that dominates the gait impairment estimation is the shanks' range of motion (RoM). This feature is related to the step length, which has been demonstrated to be levodopa responsive (57). Said property (i.e., responsiveness to levodopa) is significant, given that the main purpose of devices such as the PDMonitor^®^ is to equip physicians with the means to better evaluate symptom response to medication, and as a result have finer control over medication dose adjustments and time intake.

#### 2.2.5. Freezing of gait

Freezing of Gait (FoG) is a phenomenon described by PD patients as a sensation of their feet being “glued to the ground.” FoG is of episodic and unpredictable nature and as such, it is detected as an event, potentially with a duration of just a few seconds, rather than being considered a symptom. FoG is expressed when a patient is either shuffling forward with tiny steps, or suddenly being incapable of starting to walk, or failing to move forward. FoG can also be expressed by the complete absence of movement.

Moore et al. (58) presented a method for the calculation of an index of FoG, based on the principle that FoG is usually combined with short hesitation steps that could be detected. However, this is not always the case. A comprehensive definition of FoG such as the one used by Djurić-Jovičić et al. (59), differentiating between FoG paired with trembling and FoG paired with complete motor blocks, seems to address the problem by incorporating different types of FoG events. Nonetheless, FoG events expressed with full motor blocks are difficult to accurately detect in a home environment and they would most likely introduce a lot of false positives. Marcante et al. (60) used a system based on a pair of pressure insoles equipped with a 3D accelerometer in order to detect FoG episodes. Using it in a controlled environment they were able to report a 90% accuracy in FoG detection. The PDMonitor^®^ evaluates the presence of FoG events before the initiation of walking, during pausing phases. FoG is then detected based on the freezing index introduced by Moore et al. (58), which is estimated using data from the ankle gyroscope, as well as other features necessary for the discrimination between FoG and other kinds of activity (i.e., tremor an/or dyskinesia).

#### 2.2.6. Tremor

PDMonitor^®^ evaluates resting tremor occurring in a body segment while maintained at rest. Action (or kinetic) tremor are not evaluated by the current version of PDMonitor^®^.

Leg tremor detection is based on the activity detection method and specifically on the activities classified as “Tremor.” The activity detector is a probabilistic classifier which provides a posterior probability of a sample X belonging to a specific class, that is *P*(*Class*|*X*). The posterior probability of the activity detection classifier for the “Tremor” class, i.e., *P*(*Class* = *Tremor*|*X*) represents mainly leg activity and is averaged over a 30-minute window.

Wrist tremor assessment is based on the method presented by Cancela et al. (45), which mainly relies on the gyroscope's signal. The method consists of: signal preprocessing, tremor detection, tremor amplitude estimation and rest/posture detection. Both wrist tremor detection and amplitude estimation are based on 3-s windows. Typically, tremor has a dominant frequency on the 3.5 to 8Hz frequency band, whereas the voluntary movement frequency's range is below 2.5 − 3Hz. A number of features are extracted, including the energy of low-pass and high-pass gyroscope signals, defined, as a reference, as following:


(1) $$\begin{array}{l} En=\underset{i}{\sum }\sqrt{s_{x}^{2}\left(i\right)+s_{y}^{2}\left(i\right)+s_{z}^{2}\left(i\right)} \end{array}$$

In Equation 1, *s*_*k*_(*i*) is the *i*-th sample of the *k* axis of the signal.

A C4.5 decision tree was employed for wrist tremor detection. The wrist tremor amplitude estimation and consequently its translation to UPDRS item scores follows the approach of Rigas et al. (53) and uses a fuzzy linear function to correlate with the score of the UPDRS item 20.

#### 2.2.7. ON/OFF and fluctuations

Motor fluctuations refer to the transitions between the ON and the OFF periods. During the ON periods, medication is in effect and patients with a well-adjusted treatment plan should not experience any motor symptoms. An exception is dyskinesia, which occurs in more advanced stages of the disease. During the OFF periods, medication is not alleviating the symptoms, although is should. In advanced stages of the disease, most PD patients will experience OFF periods, with increased symptom severity, manifested unpredictably during the day.

The time during which a patient is in an OFF state is an important parameter used to assess interventions. As a result, obtaining precise information, such as the onset and the duration of OFF states, on the long term evolution of ON/OFF fluctuations is essential to optimize therapy. Currently, the only available method to collect such information is self-reported diaries. A wearable device capable of collecting PD motor fluctuations in an objective and reliable way would help overcome the limitations of those diaries and as a result would provide physicians with a valuable tool for reducing OFF periods and dyskinesia.

PDMonitor^®^ estimates the probability of a patient being in the OFF state based on a Naive Bayes classifier, taking as input the rest of the PDMonitor outputs. A feature importance technique based on the Relief method (61) is conducted in order to evaluate the importance of each feature in the detection of OFF. The features used, can be sorted into groups of interest as presented in Table 1. For the purposes of this work, a similar analysis was performed based on study data including patient diaries and UPDRS expert evaluations, in order to estimate the importance of each feature. The results for the accuracy of OFF detection are presented in Section 3.2.1.

**Table 1:** The features used by the PDMonitor^®^ for the detection of OFF, sorted into groups of interest.

**Group name**	**PDMonitor^®^**	**UPDRS items**
Activity	Lack of movement, Activity, Resting time	-
Gait	Gait, Gait with no dyskinesia	29
Tremor	Tremor score for LL, RL, LW, RW	20, 21
FoG/PI	Freezing of gait/Postural instability	14, 30
Rigidity	-	22
Body Bradykinesia	-	31, 27
Arm Bradykinesia	Bradykinesia score for LW, RW	23, 24, 25

In the last column, there is a set of UPDRS items that correspond to the same groups. LL, RL, LW, RW stand for Left/Right Leg and Left/Right Wrist, respectively.

### 2.3. Study description

**Figure 5:**
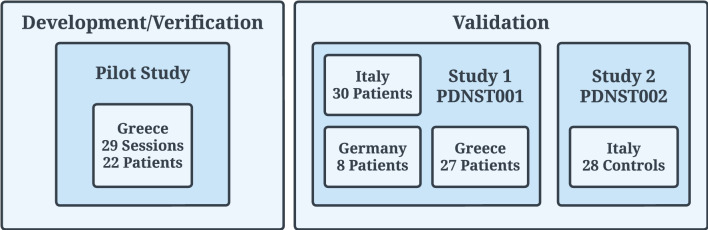
Clinical trials (Pilot, PDNST001, and PDNST002) that took place for the generation of datasets used for the development/verification and validation of the algorithms of the PDMonitor^®^ device. The PERFORM project, used for the initial development of the algorithms, is not related to the studies described in the body of this manuscript.

The data used in this work to validate PDMonitor^®^ originated from two studies (Figure 5). Specifically:

1. A study with PD patients (PDNST001) for the evaluation of the PD motor symptom assessment algorithms of the PDMonitor^®^, as well as for the wearability and usability of the PDMonitor device (Section 2.3.1).
2. A study with age-matched healthy subjects (PDNST002) for the evaluation of the wearability/usability of the device, as well as for collecting data in order to evaluate the sensitivity of the device's algorithms (Section 2.3.2).

As mentioned in Section 2.2, the data for the main development and verification of the PDMonitor^®^ algorithms were generated during a Pilot study which is described in Section 2.3.3.

#### 2.3.1. Clinical study with PD patients (PDNST001)

The PDMonitor^®^ system was used in the study PDNST001, entitled “Assessment of Parkinson's Disease's Motor Symptoms Using Inertial Measurement Devices”, in which sixty-five (65) PD patients were recruited. The total duration of the study was 5 months and it included the following two phases:

1. Phase I: Data collection for inpatients, with a participation duration not exceeding 6 h. During that time expert evaluations based on clinical scales were also regularly conducted.
2. Phase II: Data collection from continuous monitoring of outpatients at their home, or in care facilities, with a duration not exceeding seven (7) days and at least 7 h per day.

Phase II participants were a subset of patients who already participated in Phase I. The aim of the study was the assessment of PDMonitor^®^, as an integrated monitoring system for Parkinson's disease, ultimately intended to increase patients' independence, improve their quality of life and reduce the costs associated with the disease.

**Phase I**. During the Phase I of the study, the patients wore the system while staying at the hospital. At the beginning of the recording with the PDMonitor^®^, a clinical examination based on the Unified Parkinson's Disease Rating Scale (UPDRS) (62), and the Abnormal Involuntary Movement Scale (AIMS) (63) took place, preferably while patients were in an “OFF” state. If a patient was in an “ON” state, the clinical examination was postponed and rescheduled. Each patient was examined at regular intervals (30 minutes) by a physician and the whole session was recorded by a camera. The video obtained was used for the identification and evaluation of symptoms by third-party physicians (expert evaluations). The duration of the PDMonitor^®^ recording in Phase I was between 3 and 6 h for each patient. For the proper evaluation of the patients' symptoms, a diary was kept by their caregivers or nurses. Every half hour the specialized nurse or the physician asked the patient to perform specific motor tests and recorded their symptoms. During each session, patients were instructed to perform random activities that could last several minutes, for example, climbing up and down a set of stairs, making turns, lying down, standing up, walking while carrying a glass of water, carrying a heavy object, drinking a glass of water, opening and closing a door, taking a walk outside. Also there were more complex activities conducted, such as setting a table for a meal, having a meal, or even using a computer, tablet, or smartphone, etc. Normal daily activities were required in order to reduce the possible bias in symptom assessment introduced by the reduced range of patients' activity in a hospital environment.

**Phase II**. Data collected during Phase II were used to evaluate usability, validate the outcomes of the PDMonitor^®^ system vs. patient diaries as well as to evaluate day-to-day variability. The overall recording run for 1–3 days and with at least 7 h per day, whether a caregiver was present or not. Patients were trained on how to wear and use the system during their participation in Phase I.

Data from Phase I (i.e., gathered from inpatients wearing the device) were used in order to compare PDMonitor^®^ outcomes with both expert annotations (available only in Phase I) and diaries.

**Sites and Participants**. This study took place in three sites:

1. the Technische Universität Dresden (TU Dresden) in Dresden, Germany,
2. the General University Hospital of Ioannina in Ioannina, Greece and
3. the Ospedale San Camillo IRCCS, and the Padua University Hospital in Italy.

In total, sixty-five (65) PD patients were recruited. The study protocols were approved by the corresponding ethical committees and all recruited individuals signed an informed consent form. The patients' demographics are shown in Table 2.

**Table 2:** Demographics of patients participating in the PDNST001 **(top)** and PDNST002 **(bottom)** studies.

**Patient population**	
Number of participants	65
Age (Mean ± SD)	65.8 ± 9
Gender (Male/Female)	33/30
Years with PD (Mean ± SD)	8.8 ± 4.9
**Healthy population**	
Number of participants	28
Age (Mean ± SD)	63.2 ± 9.9
Gender (Male/Female)	10/19

**Scales and Questionnaires**. For the purposes of the study, the following scales and questionnaires were used:

1. Unified Parkinson's Disease Rating Scale (UPDRS) (62). A full UPDRS evaluation was conducted in the start of the session, while the Part III of the UPDRS was performed every 30 minutes.
2. Abnormal Involuntary Movement Scale (AIMS) (63). An AIMS questionnaire was filled by physicians every 30 minutes to evaluate dyskinesia exhibited by the study participants.
3. Patient/Nurse Symptom Diary (64). Diaries were filled every 30 minutes by patients, or nurses, in order to assess ON/OFF states, Dyskinesia, Bradykinesia, Tremor, FoG and general activity.
4. Comfort Rating Scale (CRS) (45). A CRS questionnaire was filled once at the end of the session in order to evaluate whether the device was comfortable to use.

#### 2.3.2. Clinical study with healthy individuals (PDNST002)

This study only included a procedure similar to that of Phase I of the PDNST001, and as such, during its course only healthy individuals (controls) used the PDMonitor^®^ device.

**Sites and Participants**. This study took place in the Ospedale San Camillo IRCCS, and the Padua University Hospital in Italy. In total, 31 healthy individuals were recruited, with data being available for 28 subjects. The healthy participants used the device for up to 3 days in a hospital environment, but they were free to move and perform any kind of daily activity, mimicking home daily living scenarios. The data resulting from this study were used mainly for evaluating the robustness of the system's algorithms in order to properly discriminate normal activities and movements from PD symptoms. The study protocols were approved by the corresponding ethical committee and all recruited individuals signed an informed consent form. The participants' demographics are shown in Table 2.

#### 2.3.3. Pilot study

The pilot study took place, chronologically, after the PERFORM project and before the PDNST001 and PDNST002 studies described herein with the purpose of data acquisition for developing the algorithms used in the PDMonitor^®^ device. The pilot study used the same protocol as the Phase I of the PDNST001 study, and it included 30 sessions performed by patients staying in the hospital between 4 and 8 h. Each session was recorded on video, and every 30 minutes a UPDRS examination (62) was performed. Moreover, a trained nurse kept a symptom diary for the entirety of each session. For monitoring the pilot study participants, a Shimmer device^1^ with 5 sensing elements was used. The sensing elements, were mounted on the ankles, wrists and the torso, in the exact same configuration as the PDMonitor^®^. The Shimmer device was used for data collection given that the PDMonitor^®^ hardware was still under development at that time.

### 2.4. Statistical analysis

#### 2.4.1. Assessment of wearability

The wearability of the device was evaluated based on the Comfort Rating Scale (CRS), filled by patients after completing the Phase II of the PDNST001 study, as well as by the control subjects of the PDNST002. The questions of the CRS are provided in Table 3. The average ratings, resulting from the responses of the patients and the control subjects, were quantitatively and qualitatively analyzed.

**Table 3:** All the questions included in the Comfort Rating Scale (CRS), a standardized questionnaire used in our work as a tool of assessing the wearability of the PDMonitor^®^ system.

**Section**	**Description**	**Controls**	**Patients**
Emotion	I feel worried and embarrassed.	0.8/20	1.9/20
	I feel tense.	0.1/20	1.8/20
	I would wear the device if it was invisible.	7.4/20	7.1/20
Attachment	I feel the device on the body.	2.3/20	3.5/20
	I feel the device moving.	1.8/20	3.0/20
	I was not able to move as usual.	0.0/20	2.6/20
	I have difficulty in putting on the device.	1.1/20	5.9/20
Harm	The attached device causes me some kind of harm.	0.0/20	0.0/20
Perceived change	I feel more bulky.	1.0/20	0.9/20
	I feel change in the way people look at me.	2.0/20	3.0/20
Movement	The device obstructs my movements.	0.3/20	2.6/20
Anxiety	I do not feel secure with the device.	0.0/20	0.5/20
	I feel that I do not have the device properly attached.	0.5/20	1.1/20
	I feel that the device is not working properly.	0.0/20	0.8/20

In the “Controls” and “Patients” columns, we present the average ratings that resulted from the responses of the control subjects of the PDNST002 study, as well as the patients of the Phase II of the PDNST001 study to the CRS questionnaire.

#### 2.4.2. Assessment of accuracy

The validation of the PDMonitor^®^ system in the identification and quantification of PD motor symptoms, as well as in the complications stemming from PD, in a statistically significant manner, is assessed based on measures of accuracy (for the detection) and measures of correlation (for the severity). Initially, the symptoms extracted through the PDMonitor^®^ were compared against the UPDRS and the AIMS scores resulting from physicians' clinical examinations, conducted in 30-minute intervals.

**Agreement with Expert on the Detection of Specific Symptoms**. For the statistical analysis, a dataset was created for each symptom, which included pairs of PDMonitor^®^ 30-minute estimations, as well as the corresponding UPDRS/AIMS items. The UPDRS/AIMS items were converted to a binary scale based on the clinical thresholds for defining a mild (or more severe) presence of a symptom. Cases with a slight symptom presence were ignored for this analysis. Then, for each symptom, an analysis based on a receiver operating characteristic (ROC) curve (65) was used to evaluate the corresponding thresholds to be set in the PDMonitor^®^. Given the thresholds obtained from the ROC analysis, a confusion matrix was computed. Accuracy, specificity and sensitivity measures were estimated and reported (Section 3.2.1).

For each symptom, specific groups of different symptoms' intensity were defined. Group differences were evaluated using the *t*-test method and box plots were generated using the Seaborn Python library (66). The created box plots are presented in Section 3. Group differences in some cases included measurements from the same patient. Therefore, patients do not belong to a specific group, neither have the same number of measurements in the same group. As a result, given this degree of variability and non-determinism, the assumption of the samples being independent, as well as the use of the t-test is justified.

**Total time estimation**. Subsequently, the thresholds indicating a significant symptom presence were employed to extract, per session, the total time of its presence, as measured by both the experts and the PDMonitor^®^ (Section 3.2.2) respectively.

A Bland Altman analysis (67) was also performed and is presented in Section 3. The intra-class correlation of PDMonitor^®^ estimation of the total time of a symptom's presence was also evaluated. To that end, the data from Phase II of the PDNST001 study were employed. The total symptom presence was estimated, for the same patient, over a number of different days, resulting in a dataset containing those estimations in pairs, forming a dataset of day-to-day symptom presence estimations. Both Pearson and Spearman correlation were used as measures of correlation and a Bland Altman analysis (67) is also reported for the bradykinesia case. The Bland Altman analysis was performed using the Matlab implementation (68). The Standard error (SSE), the Coefficient of Variation (CV) and the RPC reproducibility coefficient (1.96*SD) are included in the analysis.

**Agreement on Day-To-Day Symptom Evaluation**. For the evaluation of the day-to-day agreement of PDMonitor^®^ measures, two sets of data were used. The first, was patient data from the Phase II of the PDNST001 study, while the second, was data of healthy individuals from the PDNST002 study. The agreement was evaluated for all those patients, and control subjects (healthy individuals), having more than 1 day of monitoring activity. For each symptom, the average severity was estimated per day, and then pairs of different days were compared. Similar to the case of the total time estimation, for the day-to-day symptom evaluation, a Bland Altman analysis was performed, and both Pearson and Spearman measures of correlation were employed to evaluate the day-to-day agreement.

## 3. Results

### 3.1. Assessment of wearability

The results of the Comfort Rating Scale (CRS) for both patient and control subjects are presented in Table 3. On top of the results from the CRS questionnaire, some key findings regarding the wearability of the system, acquired through the interaction with the patients of the study, are presented below. First, it took patients about 5 minutes on average (5.3 ± 2 minutes ranging from 2 to 10.25 minutes evaluated on 39 patients), to put on all five monitoring devices (monitoring device). The procedure was recorded on video and the reported time durations were estimated based on those recordings. The wide spread in the time necessary to put on the device, was expected, and it is attributed to some patients exhibiting significant movement impairment or being in an OFF state when they were instructed to wear the device. Second, the study subjects indicated that the monitoring device worn on the waist seems to be more inconvenient compared to the devices worn on other body parts. Third, disease duration did not affect the time patients needed to put on the monitoring devices. For all patients, when comparing the time to put on the sensors to the disease duration, the Pearson's correlation coefficient (R), and its p-value, indicated that there was no significant correlation (*r* = −0.123 with *p* = 0.77, for Germany, *r* = −0.195 with *p* = 0.38, for Greece and *r* = 0.67 with *p* = 0.32, for Italy). As expected, patients who had no help putting on the sensors, needed more time than patients assisted by a caregiver (6.28 vs. 4.67 minutes).

### 3.2. Assessment of accuracy

#### 3.2.1. Agreement with expert on the detection of specific symptoms

In this section, the results regarding the agreement of the device with the expert assessments (UPDRS/AIMS evaluations performed every 30 minutes), and the symptom diaries, are presented.

**Bradykinesia**. PDMonitor^®^ arm bradykinesia estimation for 30-minute windows had significant correlation with the UPDRS arm bradykinesia subscore (*r* = 0.68) and had a rather high accuracy (0.85) in detecting patients with a sum of the bradykinesia UPDRS subscore (sum of UPDRS items 23, 24 and 25) larger than 4, as presented in Table 4. In order to further evaluate the device's performance in discriminating bradykinesia impairment, 4 bradykinesia groups were considered:

1. control individuals (referring to healthy subjects),
2. patients with 0 bradykinesia UPDRS subscore,
3. patients with < 4 bradykinesia UPDRS subscore,
4. patients with > 4 bradykinesia UPDRS subscore.

**Table 4:** Evaluation of the accuracy of PDMonitor^®^ vs. 30-min expert evaluations (UPDRS/AIMS) or diaries (for the OFF case).

**PDMonitor^®^**	**Scale item**	**Thres.^a^**	**Conf. Matrix^b^**	**Pos./Neg**.	**Acc./Spec./Sens.^c^**
Arm brad. (UPDRS)	23+24+25>4	0.7	208/41/137/781	249/918	0.85/0.85/0.84
Gait (UPDRS)	29 >1	1.6	70/39/7/903	109/910	0.99/1.0/0.67
Wrist tremor (UPDRS)	20 >1	1.64	90/17/2/2,858	107/2860	0.99/0.99/0.84
Leg tremor (UPDRS)	20 >1	0.16	28/2/1/1,440	30/1441	0.99/0.99/0.93
Dyskinesia (AIMS)	AIMS >4	1.66	68/15/9/1,607	83/1616	0.99/0.99/0.82
OFF (Diaries)	OFF	0.5	29/5/18/571	34/589	0.96/0.97/0.85
FoG (UPDRS)	14>1	0.02	10/2/1/61	12/62	0.96/0.98/0.83

The threshold derived by a ROC curve analysis (Thres.), the confusion matrix, the number of positives (true positives plus the false negatives), negatives (true negatives plus false positives), as well as the accuracy, specificity and sensitivity are also provided. The last line of the table presents the accuracy of the freezing of gait discrimination for the “Freezing” patients compared to the “No freezing” patients and the control subjects. The freezing of gait is evaluated per patient, instead of the 30-minute evaluations of the other outputs presented in this table.

a Threshold for the corresponding PDMonitor^®^ measure extracted from the ROC analysis.

b True positive/False negative/False positive/True negative.

c Accuracy/Specificity/Sensitivity PDMonitor^®^ measures are compared against specific scale items provided in the column “Scale Item.”

**Figure 6:**
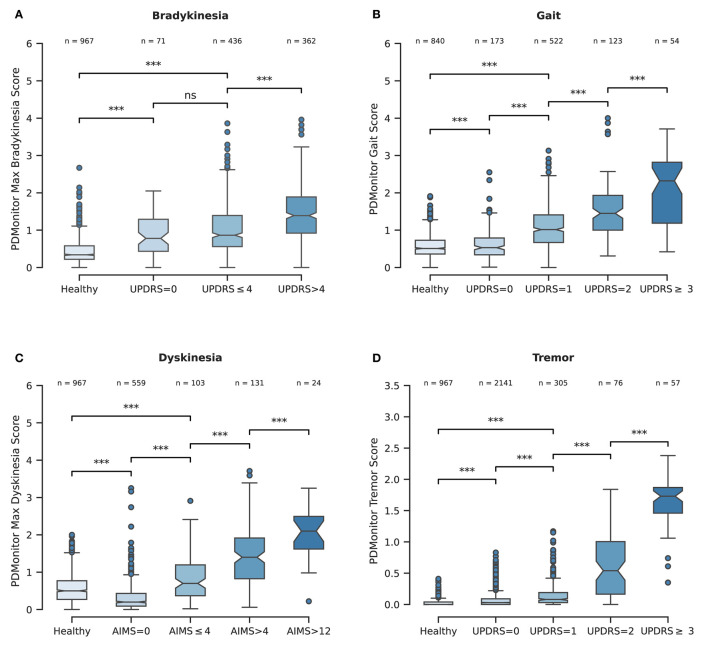
**(A)** Boxplot representing the PDMonitor^®^ bradykinesia score distribution for the different subgroups based on expert UPDRS bradykinesia evaluations. **(B)** Boxplot of PDMonitor^®^ gait score distribution for the different subgroups based on expert UPDRS gait evaluation. **(C)** Boxplot of PDMonitor^®^ dyskinesia score for the different subgroups based on expert AIMS dyskinesia evaluation. **(D)** Boxplot of PDMonitor^®^ tremor (wrist) score distribution for the different subgroups based on expert UPDRS tremor evaluation. The dots represent outliers in the dataset, while the asterisks represent statistical significance. On top of each box there is the number of data points contained within each group. Every data point represents an estimation of the respective symptom for a 30-min window. Regarding the underlying rules for the generation of the box plots, the “whiskers” extend to all points that belong within 1.5 IQR (interquartile range). The rest of the points, lying outside this range, are considered as outliers and are depicted as dots. The asterisks (*) that are drawn on top of the box plots, denote statistical significance and correspond to *p*-values' ranges. Specifically, 4 asterisks would denote *p* ≤ 0.0001, 3 asterisks *p* ≤ 0.001, 2 asterisks *p* ≤ 0.01, 1 asterisks *p* ≤ 0.05 while ns denotes *p*>0.05.

The PDMonitor^®^ bradykinesia estimation distributions for those groups are presented in Figure 6A. All groups have statistically significant different means, indicating the rather good correlation between the PDMonitor^®^ estimation and the arm bradykinesia, annotated by the experts.

**Dyskinesia**. Based on the method described in Section 2.2.2, the accuracy of the dyskinesia detection method was evaluated for the discrimination of 30 minutes' regions where the patients' AIMS score, as annotated by experts, had a value greater than 4, compared to that of those participants (control and patients) with no dyskinesia. The threshold of 4 is the minimum AIMS score for which the device can provide the most accurate results regarding the sensitivity and specificity of the detection. The accuracy obtained (Table 4) was 0.99 with an excellent specificity (0.99) and sensitivity (0.82). A high specificity is paramount, considering that the device is intended to be used in daily living and during free activities where normal movements could be confused with dyskinesia.^2^ To this end, the use of healthy subjects for the evaluation of the algorithms was rather important in order to ensure that dyskinesia can be accurately discriminated. Similarly to bradykinesia, 5 groups were considered based on their AIMS score. Those groups were:

1. control individuals (healthy),
2. patients with a 0 AIMS score,
3. patients with < 4 AIMS score,
4. patients with 4 − 12 AIMS score,
5. patients with >12 AIMS score.

The PDMonitor^®^ dyskinesia estimation distributions for those groups are presented in Figure 6C. All groups have statistically important differences indicating a rather good performance of the device in discriminating dyskinesia. It should be noted that PD patients with no dyskinesia have significantly lower dyskinesia estimations compared to both patients with slight dyskinesia (AIMS < 4) as well as healthy subjects. The only shortcoming observed with our method was the underestimation of dyskinesia in the rare case of patients having significant dyskinesia on the head or the neck and less dyskinesia in their extremities.

**Gait**. The PDMonitor^®^ gait score was evaluated for the detection of gait impairment in 30-minute windows taking into account mild and severe gait impairment according to the score of the UPDRS item 29. For the evaluation, annotations with a score of 1 in the UPDRS item 29, as well as regions with dyskinesia, were excluded. The accuracy of gait impairment detection is presented in Table 4. A rather high accuracy is achieved (0.99 accuracy with >0.99 specificity and 0.67 sensitivity). PDMonitor^®^ gait score distributions for the different expert UPDRS assessments are provided in Figure 6B.

**Freezing of gait**. During the Phase I of the PDNST001 study, 30-minute, in-clinic, sessions were annotated by experts for each patient as “Freezing” or “No-Freezing,” based on whether they identified freezing of gait in their UPDRS evaluations. The expert annotations were also compared to symptom diaries, when available. Cases where diaries and expert annotations were in disagreement were excluded, taking into account mainly cases where FoG was not observed during the UPDRS examination. It should be noted that the clinical examination included a walking test requiring the subjects to open a door and pass through it. However, the protocol neither included specific tests or activities to elicit freezing events, nor called for patients to be monitored throughout the session (recorded on video), thus limiting our ability to fully assess FoG events. As a result, the PDMonitor^®^ was evaluated in terms of discriminating between “Freezing” and “No freezing” patients based on a ROC (Receiver Operating Characteristic) analysis. To that end, first the device produced the ratio of the “*number of freezing of gait events”* compared to the “*total number of freezing of gait regions, per 30-minute periods,”* and then aggregated those ratios, per patient, for the whole session. Finally the ROC analysis for the evaluation of the discriminating power of the device was conducted. The results are presented in Table 4, in which it can be seen that the device had an excellent accuracy in the discrimination of patients exhibiting freezing of gait.

**Tremor**. Wrist tremor with a 30-minute constancy was evaluated compared to the patients' symptom diary. The accuracy of the wrist tremor detection method was initially evaluated. All 30-minute intervals with RW (right wrist) or LW (left wrist) tremor score (>1) in the UPDRS item 20 (tremor at rest) were considered as cases with tremor, whereas 30-minute windows without tremor (taking into account both the legs and the wrists) were considered as negative cases. Again, neighboring windows of different tremor classification where excluded. The confusion matrix is presented in Table 4. The specificity of tremor detection is very high (>0.99) with a significant sensitivity (>0.85). Based on the method described in the corresponding part of Section 2.2.6, the accuracy of leg tremor was also evaluated. Accuracy, sensitivity and specificity, along with the confusion matrix are presented in Table 4. The accuracy of the PDMonitor^®^ in the discrimination between those patients that exhibit more than slight leg tremor compared to those patients that exhibit no tremor in 30-minute intervals is 0.99. As presented in Figure 6D the device is able to accurately discriminate tremor rated with a UPDRS item 20 score of >1. It should be noted that neighboring samples with different UPDRS annotations were not excluded in the box-plot and therefore the overlapping between the distributions could be even smaller in practice.

**Figure 7:**
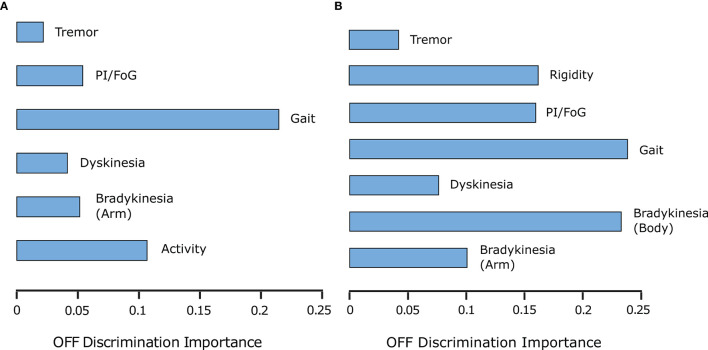
**(A)** PDMonitor^®^ output importance for OFF detection (per group defined in Table 1). **(B)** UPDRS item importance for OFF detection (per group in Table 1).

**ON/OFF and Fluctuations**. PDMonitor^®^ OFF estimation is based on a method combining the individual symptoms and measures produced by the device. The results of the Relief method for assessing the importance of each symptom in estimated OFF periods are presented in Figure 7A. As discussed in Section 2.2.7, a similar analysis was performed trying to estimate OFF periods as were reported in symptom diaries. The results are presented in Figure 7B for PDMonitor^®^ and UPDRS annotations respectively. Features related to gait, postural instability and gait difficulties (PIGD) have the highest importance in discriminating between ON and OFF states consistently in both the PDMonitor^®^ and the UPDRS estimations. The UPDRS body bradykinesia (UPDRS items 27 and 31) had similar importance with gait. However, this was expected since the correlation of gait (UPDRS item 29) with the rising from chair activity (UPDRS item 27) in our study was very high (*r* = 0.88). Therefore, the order of the symptoms' importance is consistent between the PDMonitor^®^ and the expert annotations, highlighting again the rather good agreement between the device and the expert raters. The accuracy, the sensitivity and the specificity of the OFF score produced from the PDMonitor^®^ compared to the UPDRS annotations and the symptom diaries was evaluated in 30-minute windows and is presented in Table 4. The evaluation included exclusively 30-minute intervals where estimations for gait from the PDMonitor^®^ were available. It should be noted that for one site, diaries were not filled in during the Phase I of the PDNST001 study. Moreover, neighboring 30-minute intervals with different OFF evaluations where excluded, in order to reduce possible errors due to the transition between OFF and ON states (and vice versa). The accuracy and the specificity of the OFF detection method was excellent (0.96 and 0.97 respectively).

#### 3.2.2. Agreement on the total time of presence of specific symptoms

**Figure 8:**
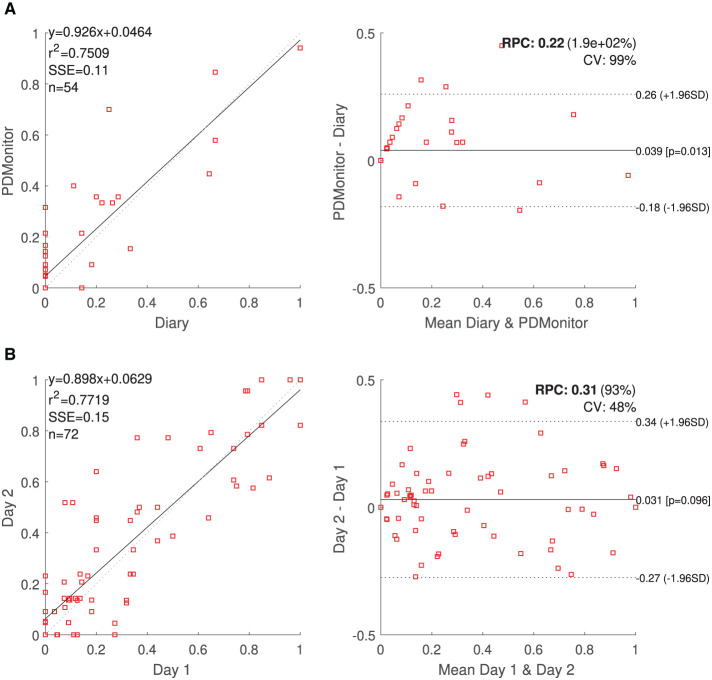
**(A)** PDMonitor^®^ OFF estimation and Bland Altman plot for the patient diaries. **(B)** Correlation and Bland-Altman plots for day-to-day agreement of PDMonitor^®^ estimated time percentage, where (left leg) bradykinesia score was more than 1 (UPDRS).

**OFF Time**. For each session, the percentage of each patient in the OFF state was calculated. The percentage of time while a patient was in the OFF state was estimated as the ratio of the “*number of evaluations where the probability for being in an OFF state was higher than* 0.55*,”* to the “*total number of evaluations.”* A high correlation, *r*^2^ = 0.75, between the PDMonitor^®^ estimations and the combination of UPDRS evaluations and symptom diaries is observed, as it can be seen in Table 5. Correlation and Bland-Altman plots are presented in Figure 8A.

**Table 5:** Correlation of the measures “Time with OFF” and “Time with dyskinesia,” as were estimated by the PDMonitor^®^ system, compared to expert annotations (and diaries in the case of “Time with OFF”) per recording/session.

**PDMonitor^®^**	**No. of patients**	**Correlation (*r*^2^)**	**Spearman's Rho**
Time with OFF	54	0.75	0.65
Time with dyskinesia	80	0.63	0.77

The “Time with OFF” was calculated from expert annotations using the ratio of the “*number of evaluations where the probability for being in an OFF state was higher than* 0.55”, to the “*total number of evaluations.”* ‘Time with dyskinesia”. On the other hand, the “Time with dyskinesia” was calculated as the percent of expert annotations in which the AIMS scores were higher than 4.

**Dyskinesia Time**. The time with Dyskinesia is estimated in a similar fashion, considering the percent of expert annotations in which the AIMS scores were higher than 4. A total of 80 subjects were included in this analysis and the correlation of the variable called “Time with dyskinesia” as produced by the PDMonitor^®^, compared to the expert assessments was *r*^2^ = 0.63 (Table 5).

#### 3.2.3. Agreement on day-to-day symptom evaluation

The day-to-day agreement was evaluated for those patients of PDNST001, Phase II and those controls (healthy individuals) of PDNST002, having more than 1 day of monitoring activity. For each symptom, the average severity was estimated per day, and then pairs of different days were compared. Correlation and Bland-Altman plots of the results are presented, for bradykinesia, in Figure 8B. The ICC correlations are presented in Table 6. Considering the fact that there is an intrinsic variation in the PD symptoms, the device's ICCs could be considered rather high.

**Table 6:** Intra-class correlation coefficients of PDMonitor^®^ estimated measures for different recording days.

**PDMonitor^®^**	**ICC (*r*^2^)**	**Spearman's Rho**
Time spent with bradykinesia (LL) >1	0.77	0.83
Time spent with dyskinesia (LL) >1	0.82	0.45
Time spent with gait >1	0.71	0.83

This analysis evaluated the day-to-day agreement for those patients of PDNST001, Phase II and those controls (healthy individuals) of PDNST002, having more than one day of monitoring activity. For each symptom, the average severity was estimated per day, and then pairs of different days were compared.

## 4. Remarks

During the course of the first study (PDNST001), the UPDRS annotations were performed by one physician, although different in each site. Diaries were not filled in one site during the Phase I of the PDNST001 study and as a result, for this site, we were not able to compare the UPDRS evaluations with the corresponding diaries. The majority of the recordings were performed inside hospitals, but the subjects were free to perform any kind of activity. For example, in one site, many patients also performed rehabilitation exercises. Therefore, even if the recordings were not in the patients' actual environment, the conditions during the studies were quite close them. Moreover, since patients are instructed not to use the device during intense activities, the actual conditions they encounter during their everyday lives, while wearing the PDMonitor^®^, were expected to be quite similar to the ones they experienced during the studies. Intense activities were defined as any activity other than walking. The patients were advised not to wear the monitoring devices during intense activities as there would be abrupt signal changes logged by the IMU sensors, which would contaminate the system's output.

## 5. Discussion

PDMonitor^®^ is a monitoring system that has been developed for the detection and follow-up monitoring of parkinsonian symptoms based on wearable monitoring devices. Although, it should be noted, that the device does not replace, neither a clinical examination, nor a patient's symptoms report, and any findings should be always verified with the patients and their caregivers. The aim of the studies presented herein, was to validate the system's usability and efficacy in the detection of motor symptoms manifested in Parkinson's disease.

The first significant outcome of the PDNST001 study was the confirmation that PDMonitor^®^ can be effectively, and easily, used by patients and caregivers. As a reference, in order to use the system, about 5 minutes are required, in average, for mounting all sensors, although patients in the OFF state may need more time or additional help. This finding is also confirmed by the CRS questionnaire in which the question “I have a difficulty in putting on the device” received a higher score by the patients compared to the control subjects. It should be noted that the PDMonitor^®^ device has a number of features that enable its unsupervised use in hospital and home environments. The first important feature, is the ability to automatically identify the position of each of the 5 monitoring devices on a patient's body, thus, significantly reducing the complexity/burden of wearing the 5 sensors, as well as the probability of device misuse. Another important feature is that no user interaction is needed to start a recording, apart from undocking and wearing the monitoring devices, as well as putting them back overnight for data transfer and charging. A point to note is that, in terms of usability, the question “I would wear the device if it was invisible” of the CRS, received an increased score. This question is probably answered by patients having in mind the stigma around medical conditions, and thus it denotes a wish for discreet “invisible” medical devices in general. As a result, this is a well known aspect of similar devices (45) and PDMonitor^®^ design aims to reduce such concerns. More data from real world use may be needed to further evaluate the effect of such issues on the usability of the device. The effective use of the system was also demonstrated in the study performed by Bendig et al. (69), where 12 subjects used the monitoring devices for 3 months and demonstrated significant adherence and satisfaction (both being prerequisites for effective use).

The second major outcome of the study is related to the performance of the device in the detection of PD related motor symptoms. Statistical analysis comparing the symptoms detected by the PDMonitor^®^, to those identified through clinical evaluation and patient diaries, revealed the system's capacity to accurately detect the majority of PD motor symptoms and their fluctuations. Table 4 summarizes all PDMonitor^®^ outcomes and their accuracy measures, compared to the detection and severity estimation of PD motor symptoms based on the expert evaluations or diaries. In all cases, the outcomes of the PDMonitor^®^ algorithms were translated to clinically relevant scales which are familiar to movement disorders healthcare professionals, aiming to immediately offer actionable knowledge. Even in cases where the accuracy was moderate, the specificity was very high. This was an important requirement of the device, considering the fact that it is intended to be used at home, as well as in general unconstrained, environments with a need of avoiding false positives occurring during daily activities. The significant day-to-day correlation between symptoms presented in Section 2.4.2 is also very important as it depicts the repeatability of the device's outcomes. This is also further supported by the fact that both bradykinesia and gait impairment were statistically different between control subjects (healthy individuals) and PD patients with a UPDRS score of 0 on the respective UPDRS items (Figures 6A, B). The results for both OFF and dyskinesia time estimation are also very important (*r*^2^ = 0.75 and *r*^2^ = 0.63 respectively) considering the sparse evaluations (30-minute intervals) and a typical duration of each session between 4 and 8 h. Therefore, PDMonitor^®^ provides a rather comprehensive, and accurate, evaluation of the main parkinsonian symptoms. Each one symptom worths a further evaluation, in greater technical and clinical detail, in which there will be also presentations of specific cases. However, this was not possible in the context of this work due to space limitations. We will focus on this task in a future work.

Moreover, there are specific cases where the limitations of the physical examination were highlighted, even though they were not systematically evaluated in the studies. For example, some patients did have significant altered symptom manifestations before and during the clinical examination, including gait difficulty, which was however clearly depicted in the PDMonitor^®^ report as it is not based only on a specific time period in which the symptoms may have subsided. This further supports the need of using remote monitoring in clinical practice. Also, a very interesting fact is that features related to gait, postural instability and gait difficulties (PIGD) seem to be better indicators of OFF, compared to arm bradykinesia. This may further highlight the importance of ambulatory gait evaluation for assessing PD patient monitoring.

The progression of the neurodegeneration process in PD is related to the emergence of motor complications, such as fluctuations and dyskinesia, which are often difficult to predict and manage, especially in advanced patients (14, 70). The treatment strategies that are currently available for PD, as it advances, include lifestyle changes, fine tuning of oral medication, different routes of drug administration, and deep brain stimulation (13, 71). However, the efficacy of these treatments is limited and it relies mainly on the information that physicians manage to acquire regarding each patient's symptomatology, which does not always depict with accuracy the patient's overall state and disease progression. A study performed by Erb et al. (72) found that 38% of all participants who were asked to complete an electronic motor diary at home missed approximately 25% of all possible entries. Also, the entries the participants made had an average delay of more than 4 h. During clinical evaluations by PD specialists, self reports of dyskinesia were marked by approximately 35% false negatives and 15% false positives. Compared to the live examinations, the video evaluations of the Part III of the UPDRS significantly underestimated the subtle features of tremor and extremity bradykinesia, suggesting that these aspects of the disease may be misjudged during remote assessments. On the other hand, based on the results of this study, PDMonitor^®^ can effectively detect the majority of PD related motor symptoms, with high test-retest reliability. The device also provides a highly accurate estimation of OFF and dyskinesia time, which is crucial for any therapeutic decision.

Other systems previously reported to detect parkinsonian symptoms in PD (36, 73, 74), do provide useful information to physicians leading to improved therapeutic decisions and patient outcomes (41, 75). However, PDMonitor^®^ has the main advantage of evaluating all motor symptoms and their complications, including gait, freezing of gait and postural instability. The detection of freezing of gait along with other problems related to postural instability and gait difficulties (PIGD) is a key component when we try to optimize pharmacological and non-pharmacological treatment in Parkinson's (13, 71). These symptoms also have a strong effect on a patient's quality of life. The recent COVID-19 pandemic has further highlighted the importance of telemedicine and remote monitoring as a way to hamper the impact of social and mobility restrictions, particularly in patients in advanced stages of the disease and those that have undergone invasive treatments (19, 76).

PDMonitor^®^ is designed for long-term continuous monitoring, enabling a new paradigm in PD management. Long-term and continuous monitoring facilitates the early detection of fluctuations (wearing off) and PIGD in patients, which the treating physicians could not otherwise identify. Timely detection and treatment could help patients better understand their status (77) and improve the probability of living a normal life while staying effective in their work. This is expected to have a serious impact to the Health Economics of the System and the patients' Quality of Life. Tsamis et al. (22) presented two specific cases where the potential of PDMonitor^®^ to accurately capture the diverse clinical manifestations of advanced PD was demonstrated, thus reducing the need for prolonged in-person examinations or hospitalization. Both presented cases, included significant difficulties in the diagnostic approach, due to missing information regarding the time course of symptoms throughout the day. With the use of PDMonitor^®^, physicians had access to an objective assessment of the patients' motor symptoms, as these were manifested in their daily home environments, managing to reach a final diagnosis and making the right treatment decisions.

PDMonitor^®^ also offers the possibility to be used for advanced therapy selection based on a set of patient eligibility criteria. For example, Antonini et al. (9) have developed a screening tool for identifying patients eligible for deep brain stimulation (DBS). The tool consists of a number of questions regarding PD motor symptoms and their fluctuations, such as:

- *Do you have* ≥2 *h of OFF time per day?*
- *Do you experience unpredictable fluctuations?*

Objective measurements and measures like the ones suggested, based on PDMonitor (78), may complement such screening tools and provide a valuable instrument for a timely and accurate patient selection eligible for advanced therapies.

Furthermore, PDMonitor^®^ can be used for post-DBS monitoring and tuning. The challenge in post-DBS management is to find the proper stimulation paradigm along with the proper medication treatment. The problem increases when the patients go home and after 3–4 weeks they start losing the acute effect of their therapy, creating the need for further medication optimizations. This is a use case when a medical device, like PDMonitor^®^, could be really useful, as it can guide the medication adjustment through precise monitoring, fulfilling a true unmet need of moving the patients' care away from the hospital and to the home.

Dorsey et al. (79) also supported that in order to improve PD care, more of it must be delivered at home. Emerging care models will combine remote monitoring, self-monitoring, and multidisciplinary care in order to enable the provision of patient-centered care at home and decrease the need for in-clinic assessments. It should be noted that PDMonitor^®^ also provides an accompanying mobile app with important features like medication and medication intake, as well as a symptom diary. All logged information is also available in the PDMonitor^®^ reports as those presented in Figures 2A, B. The mobile app also includes educational material and provides to each patient a form of an one-way communication with their physician. It is known that mHealth solutions tend to increase patient awareness and disease self-management, as demonstrated in similar applications (80). Therefore, based on the results of the studies (PDNST001 and PDNST002) and considering the usability, the performance and the clinical need, PDMonitor^®^ could be considered as a tool that could be essential in daily practice and in the management of Parkinson's disease. New and ongoing studies are expected to provide additional evidence about the clinical benefits of this new paradigm, that PDMonitor^®^ is a part of, enabling a wider adoption (81). Physicians and healthcare systems may need to adopt and embrace this new paradigm in order to overcome current barriers (77, 82) as well as to unlock the full potential of continuous patient monitoring.

## 6. Conclusions

Objective symptom monitoring in Parkinson's disease can be a groundbreaking tool for the proper management of the disease and the therapeutic decision making process. Monitoring the most important PD motor symptoms with high accuracy, may contribute to better, more precise and more effective treatment interventions. The results of these studies demonstrated that PDMonitor^®^ can provide a comprehensive evaluation of the majority of motor symptoms, with significant accuracy, as compared to expert assessments and patient/caregiver diaries, and also that it can be easily used by the patients and their caregivers. PDMonitor^®^ enables longitudinal objective monitoring of patient symptoms and their lifestyle, unlocking important patient management potential.

## Related patents

The following patent has been filed and published: WO2020120999A1 Monitor system of Multiple Parkinson's Disease Symptoms And Their Intensity.

## Data availability statement

The datasets presented in this article are not readily available due to being property of PD Neurotechnology Ltd. Requests to access the datasets should be directed to PD Neurotechnology Ltd. (info@pdneurotechnology.com).

## Ethics statement

The studies involving human participants were reviewed and approved by the Ethical Committee of the TU Dresden University, the Ethical Committee of the University Hospital of Ioannina and the Ethical Committee of the IRCCS San Camillo Research Hospital. The participants were fully informed about all aspects of their participation in the studies and provided a written informed consent form.

## Author contributions

AA, HR, and SK: study design, conceptualization and supervision, as well as patient enrolment. BF, AF, and KT: manuscript review and editing. GG, MG, and CT: study conceptualization, patient enrolment, data collection, curation, and validation. GR: study design, original draft preparation, statistical analysis, and data visualization. NK: original draft preparation and manuscript review. AN: manuscript review and editing, as well as data visualization. CP: manuscript writing, review and editing. All authors approved the submitted version of the manuscript.

## Footnotes

^1^ http://www.shimmersensing.com

^2^It should be noted that according to the device's instructions for use, the device is not intended to be worn during intense activities (i.e., any activity other than walking). The reason being, signals logged by the IMU sensors during intense activities would contain abrupt changes that would contaminate the system's output.
